# Entropy Derived from Causality

**DOI:** 10.3390/e22060647

**Published:** 2020-06-10

**Authors:** Roland Riek

**Affiliations:** Laboratory of Physical Chemistry, ETH Zuerich, Wolfgang-Pauli-Strasse 10, HCI F 225, CH-8093 Zurich, Switzerland; roland.riek@phys.chem.ethz.ch

**Keywords:** discrete time, causality, entropy

## Abstract

The second law of thermodynamics, with its positive change of entropy for a system not in equilibrium, defines an arrow of time. Interestingly, also, causality, which is the connection between a cause and an effect, requests a direction of time by definition. It is noted that no other standard physical theories show this property. It is the attempt of this work to connect causality with entropy, which is possible by defining time as the metric of causality. Under this consideration that time appears only through a cause–effect relationship (“measured”, typically, in an apparatus called clock), it is demonstrated that time must be discrete in nature and cannot be continuous as assumed in all standard theories of physics including general and special relativity, and classical physics. The following lines of reasoning include: (i) (mechanical) causality requests that the cause must precede its effect (i.e., antecedence) requesting a discrete time interval >0. (ii) An infinitely small time step dt>0 is thereby not sufficient to distinguish between cause and effect as a mathematical relationship between the two (i.e., Poisson bracket) will commute at a time interval dt, while not evidently within discrete time steps Δt. As a consequence of a discrete time, entropy emerges (Riek, 2014) connecting causality and entropy to each other.

## 1. Introduction

Causality is the connection between causes and effects [1]. Itis considered to be fundamental to all natural sciences including physics as well as philosophy and has therefore been investigated by both faculties extensively (as summarized for example by Bunge [1]). Within the realm of physics mechanical causality requests the principle of antecedence, which means that an effect cannot occur from a cause that is not in the past. In special relativity theory, this statement is further constrained and requests that an effect cannot occur from a cause that is not in the past light cone of that event. This description links causality and time close together and requests a time interval between cause and effect [2]. Furthermore, time can only be measured if there is a relationship between cause and effect as in a clock, which is the device of time measurement. This dual relationship has been already described by Aristoteles: “Not only do we measure the movement by the time, but also the time by the movement, because they define each other.” [3]. Based on this argumentation and the finding that causality is absolute but time relative in special relativity and that time order is invariant under Lorentz transformation only if the events in question can be connected by signals (i.e., causal chains) [4], we state that causality is more fundamental than time as also expressed by Reichenbach with the wording that “time order is reducible to causal order” [4]. Furthermore, we define here time as the metric of causality. With this definition of time it is demonstrated in the following that time must be discrete in nature. One of the many consequences thereof [5,6] and of interest here is that entropy emerges if time is discrete in nature but treated to be continuous [5]. Hence, it appears that casualty with its arrow of events and entropy with its arrow of time are closely related.

The structure of the presented work starts with the derivation of the necessity of a discrete time if time is the metric of causality (Section 2.1, Section 2.2, Section 3.1 and Section 3.2). Some of the consequences of a discrete time (i.e., entropy) are then described in paragraph 3 followed by a discussion (Section 4) and a conclusion (Section 5).

## 2. From Causality to a Discrete Time

### 2.1. Causality and Time

Causality is a common phenomenon within our daily experience. It is the relationship between a cause and its effect. Causality describes therefore the process and consequences from an action (please note, this is not the action used in physics). Its presence is requested here as an axiom as has been done in classical physics [7] as well as special and general relativity theory [8,9]. It is further stated that the relationship (i.e., process) between the cause and its effect can be described accurately with a function *C*, which is position or/and momentum-dependent. It is evident that this description called by Bunge [1] mechanical causality is deterministic and requests the presence of space as a causal change of a system can only happen within space. Furthermore, it requests antecedence of the cause in respect to the effect [2]. Time *t* is defined here as the metric of causality which permits the process to be described with a function C(▵t) with ▵t the time between the cause and the effect to fulfill the requested antecedence. Importantly, the time-dependent change must have a time direction to distinguish between the origin of causation and the effective result caused. The function describing causality is therefore requested to be of unidirectional nature and not of bidirectional nature (i.e., bijective nature) because a given result may originate from distinct processes and cannot turned around and because of the lack of the possibility to go backward in time by human experience.

In general, the evolution of this function *C* from the cause, to the effect at time ▵t is given by

(1) C(▵t)

At infinitely small time steps with

(2) $$lim_{▵t\;\to 0}C\left(▵t\right)=C\left(0\right)=0$$

(with C(0)=0 by definition). With other words, at infinitely small time steps there is no causality because the cause and the effect cannot be distinguished from each other. It is noted that this argumentation parallels Zeno’s 3. paradox that a flying arrow does not move since at any instant it is motionless (which paradox could be solved by infinitesimal calculus). In contrast to Zeno however, the subject of interest is not the velocity but the causality. This suggests that time cannot be continuous if it is the metric of causality and thus time must be discrete in nature.

Next, we shall get concrete by defining the function *C*. We start with a classical description of the Hamilton function H(q,p) with $q=(q_{1},q_{2},q_{3})$ and $p=(p_{1},p_{2},p_{3})$ for the generalized phase space canonical position and momentum, respectively, and the acting potential V(q) (in cartesian coordinates the Hamilton function reads $H\left(q,p\right)=\frac{1}{2}mp^{2}+V\left(q\right)$) [10]. Using the Hamilton equation ${\dot{p}}_{i}=-\frac{\partial H}{\partial q_{i}}$ the second law of Newton is obtained [10]

(3) $$-\frac{\partial V}{\partial q_{i}}=lim_{▵t->0}\left(\frac{p_{i}\left(t+▵t\right)-p_{i}\left(t\right)}{▵t}\right)$$

The function C(▵t) is then defined by $-\frac{\partial V}{\partial q_{i}}▵t$ through (see also below around Equation (10))

(4) $$p_{i}\left(t+▵t\right)=-\frac{\partial V}{\partial q_{i}}▵t+p_{i}\left(t\right)$$

connecting the cause described by the force $-\frac{\partial V}{\partial q_{i}}$ that acts during the time ▵t on p(t) with the effect described by p(t+▵t), respectively (please note, the variable *t* is arbitrarily chosen). With a force (ready to cause) acting during an infinitely small time step (i.e., $lim_{▵t->0}▵t=0$) there is no effect. That means, a force can only have an effect if time is of discrete nature since a discrete value cannot be equal to 0. A critical reader may state that the time step can be infinitely small but larger than 0 and must does not be of discrete nature. It is in our definition of an infinitely small time step that it approximates 0 at the limit ($lim_{▵t->0}▵t=0$) which is actually done in the standard description of the second law (Equation (3)) without hesitation because the second law of Newton is a continuous function yielding acceleration. We thus request time to be of discrete nature.

To further strengthen our argumentation on the impact-full difference between ▵t>0 and ▵t=0 we shall now fundamentally elaborate on the relationship between the cause and effect and the role of a discrete time step using the following mathematical law: If two functions f(q,p) and g(q,p) “commute” within the Poisson bracket with $\left\{f,g\right\}=\sum_{j=1}^{n}\frac{\partial f}{\partial q_{j}}\frac{\partial g}{\partial p_{j}}-\frac{\partial f}{\partial p_{j}}\frac{\partial g}{\partial q_{j}}=0$ (and *n* the number of degrees of freedom) they are in involution with each other and thus they are independent of each other (i.e., do not have the same degree of freedom) [10]. With other words if $\{p_{i}\left(t+▵t\right),-\frac{\partial V}{\partial q_{i}}▵t\}=0$ there is no causality possible because there is no connection between them. Indeed, as shown below $\{p_{i}\left(t+▵t\right)$ and the change/the cause $-\frac{\partial V}{\partial q_{i}}▵t$ are in involution if and only if ▵t is infinitely small:

(5) $$\left\{p_{i}\left(t+▵t\right),-\frac{\partial V}{\partial q_{i}}▵t\right\}=\sum_{j=1}^{n}\frac{\partial p_{i}\left(t+▵t\right)}{\partial q_{j}}\frac{\partial -V}{\partial p_{j}\partial q_{i}}▵t-\frac{\partial p_{i}\left(t+▵t\right)}{\partial p_{j}}\frac{\partial -V}{\partial q_{j}\partial q_{i}}▵t$$

$$=\sum_{j=1}^{n}\frac{\partial (-\frac{\partial V}{\partial q_{i}}▵t+p_{i}\left(t\right))}{\partial q_{j}}\frac{\partial -V}{\partial p_{j}\partial q_{i}}▵t-\frac{\partial (-\frac{\partial V}{\partial q_{i}}▵t+p_{i}\left(t\right))}{\partial p_{j}}\frac{\partial -V}{\partial q_{j}\partial q_{i}}▵t$$

$$=\sum_{j=1}^{n}\frac{\partial (-\frac{\partial V}{\partial q_{i}}▵t)}{\partial q_{j}}\frac{\partial -V}{\partial p_{j}\partial q_{i}}▵t-\frac{\partial (-\frac{\partial V}{\partial q_{i}}▵t)}{\partial p_{j}}\frac{\partial -V}{\partial q_{j}\partial q_{i}}▵t-\frac{\partial -V}{\partial q_{j}\partial q_{i}}▵t$$

$$=\sum_{j=1}^{n}\frac{\partial^{2}V}{\partial q_{j}\partial q_{i}}▵t$$

With an infinitely small ▵t: $\lim_{▵t->0}▵t=0$

(6) $$lim_{▵t->0}\left\{p_{i}\left(t+▵t\right),-\frac{\partial V}{\partial q_{i}}▵t\right\}=0$$

This means that if and only if ▵t>0 the Poisson bracket is different from zero ($\{p_{i}\left(t+▵t\right),-\frac{\partial V}{\partial q_{i}}▵t\}\neq 0$) and therefore we argue that there is only causality if time is of discrete nature. The cause during the time step ▵t (i.e., $\frac{\partial V}{\partial q_{i}}▵t$) can thus have an effect (Equation (4) in the presence of a non-trivial potential V(q). With the introduction of a discrete time also a time direction [5] is obtained in contrast to the guaranteed time reversibility of the second law of Newton through its second order time derivative along with its infinitely small time step [4]. This can be illustrated by going a causality event forward with Equation (4) in a first step and then going a step backward with $p_{i}\left(t+▵t\right)$ the starting state and the effect (result) given by $p_{i}\left(t\right)$ which yields

(7) $$p_{i}\left(t\right)=\frac{\partial V}{\partial q_{i}\left(t+▵t\right)}▵t+p_{i}\left(t+▵t\right)=\frac{\partial V}{\partial q_{i}\left(t+▵t\right)}▵t-\frac{\partial V}{\partial q_{i}\left(t\right)}▵t+p_{i}\left(t\right)$$

This equation is only true for an infinitely small time step $\lim_{▵t->0}▵t$ or a trivial potential V(q). In conclusion, time must be of discrete nature, if it is considered to be the metric of causality. As a consequence of a discrete time, there is an arrow of time [4], which of course is requested by causality.

### 2.2. Under a Dynamic Discrete Time

In this section, a physical non-relativistic classical theory is established with a discrete time using the dynamical discrete time approach by Lee [11].

If time is a discrete dynamical variable $t_{n}$ the continuous space function q(t) of a system is replaced by a sequence of discrete values $q_{n}=q\;\left(t_{n}\right)$ and $p_{n}=p\;\left(t_{n}\right)$ with [11]:

(8) $$\left(q_{0},p_{0},\;t_{0}\right),\;\left(q_{1},p_{1},\;t_{1}\right),\ldots ,\;\left(q_{n},p_{n},\;t_{n}\right),\ldots ,\;\left(q_{N+1},p_{N+1},\;t_{N+1}\right)$$

with $\left(q_{0},p_{0},\;t_{0}\right)$ the initial and $\left(q_{N+1},p_{N+1},\;t_{N+1}\right)$ the final “position”. In this description $q_{n}$ and $p_{n}$, are still continuous, while $t_{n}$ is of discrete character and is only changing if there is causality, i.e., an acting force which requests $p_{n}\neq p_{n-1}$ during a time $\Delta t_{n}$. Under these circumstances $\Delta t_{n}=t_{n}-t_{n-1}\;$, which is dynamic in nature if a time reversible/symmetric descriptions is requested that can approximate the continuous time theories or alternatively is constant $\Delta t_{n}=\Delta t$ if a time irreversible description is permitted/requested.

The Hamiltonian function at point *n*$H_{n}\left(q_{n},p_{n}\right)$ follows the discrete Hamiltonian equation [5]:

(9) $$\frac{1}{\Delta t_{n}}\left(p_{i,n+1}\left(t_{n}+▵t_{n+1}\right)-p_{i,n}\left(t_{n}\right)\right)=-\frac{\partial H_{n}}{\partial q_{i,n}}$$

yielding the discrete second law of Newton but written differently:

(10) $$p_{i,n+1}\left(t_{n}+▵t_{n+1}\right)=-\frac{\partial V}{\partial q_{i,n}}▵t_{n}+p_{i,n}\left(t_{n}\right)$$

The latter is the descriptor for causality with the cause on the right (i.e., with the acting force during a time step) “working” on the system (i.e., the momentum at point *n*) and the effect on the left (i.e., the new momentum at point n+1).

The space and momentum variables are dependent on each other as follows

(11) $$▵t_{n}=m\frac{q_{i,n}-q_{i,n-1}}{p_{i,n}}$$

if and only if $p_{i,n}\left(t_{n-1}+▵t_{n}\right)\neq p_{i,n-1}\left(t_{n-1}\right)$ and $p_{j,n}\left(t_{n-1}+▵t_{n}\right)\neq p_{j,n-1}\left(t_{n-1}\right)$ with j≠i and a carefully selected reference frame (i.e., such that the force acts only in one direction and such that $p_{i,n-1}=0$). Please note that the last equation corresponds to a definition of the discrete momentum (i.e., $p_{n}=lim_{\Delta t_{n}-0}\;m\frac{q_{n}-q_{n-1}}{\Delta t_{n}\;}$) [4] but written differently because it is used to define the time step. It is thereby important to understand that there is no time step unless the momentum changes—actually in absence of a momentum change the time step is then infinitely large (with $\lim_{p_{i,n}\to p_{i,n-1}}▵t_{n}\to \infty$). If the system moves with constant velocity (or constant momentum) there is no time as there is nothing caused. At the moment of a momentum change, a time step is realized following the above equation. The causality relationship of Equation (8) is then given by

(12) $$p_{i,n+1}=-\frac{\partial V}{\partial q_{i,n}}m\frac{q_{i,n}-q_{i,n-1}}{p_{i,n}}+p_{i,n}$$

In the absence of a force (i.e., $\frac{\partial V}{\partial q_{i,n}}=0$) the momentum does not change, as expected. In the presence of a force, the momentum changes in dependent of past (i.e., at point n−1) and present (i.e., at point *n*) information yielding the future momentum at point n+1 [5]. Time as such is not present anymore. There are only points (depicted by n,n−1,n+1) that can be interpreted as states, which change yielding a dynamic, however. In this description, it gets evident that a system with momentum and space evolves from state to state (Equation (12)) without the request of a time variable showing that time is not fundamental. This finding parallels with the description of general relativity by the Hamilton-Jacobi formulation, which is an equivalent formulation of standard general relativity, but without an explicit time [12]. As stated by Rovelli, in the latter description many physical variables change together, and no preferred clock or parameter is needed to track change. In such a dynamic albeit no explicit time, there is a “becoming” using a word by Rovelli, which in the case presented is described by the causality

(13) $$C_{n\to n+1}=-\frac{\partial V}{\partial q_{i,n}}▵t_{n}=-\frac{\partial V}{\partial q_{i,n}}m\frac{q_{i,n}-q_{i,n-1}}{p_{i,n}-p_{i,n-1}}$$

During the step n−1 to *n* the force $-\frac{\partial V}{\partial q_{i,n}}$ is generated yielding a causal event from state n→n+1.

Time as a metric gets only useful, if one array of states is compared with another one (for example a clock). The time step between two states of causality is then given by Equation (13). The time step $▵t_{n}$ is thereby related to the velocity relative to the acceleration at state *n*. It seems obvious that this time description can be relative as found in special relativity. It makes sense to distinguish between two scenarios: (i) a discrete time reversible description with its limit $lim_{▵t_{n}->0}$ approximating a time continuous case and (ii) an irreversible description with $▵t_{n}=▵t$. In the reversible case $▵t_{n}=s_{n}▵t$ with $s_{n}$ the scaling factor as used in [5]. In this case, there is a reversibility axiom requesting $\frac{s_{n}}{s_{n+1}}=\frac{F_{i,n+1}}{F_{i,n}}$ [5] (see also below). This yields

$$C_{n\to n+1}=-\frac{\partial V}{\partial q_{i,n}}▵t_{n}=F_{i,n}s_{n}▵t=F_{i,n+1}s_{n+1}▵t=C_{n+1\to n+2}=const$$

and with this

(14) $$▵t_{n}\propto \frac{1}{F_{i,n}}$$

Hence, causality is constant, and time is dynamic as indicated by Lee [11]. Reversibility is gained because causality *C* is constant. The forward causal event n→n+1 can be reversed by the backward causal process n+1→n ($C_{n\to n+1}+C_{n+1\to n}=0$).

In standard microscopic physical theories this approach is used with infinitely small steps losing the causality argument per se. It is, however, known that this is not a correct approach since thermodynamics states the irreversibility of nature. For this scenario (ii) $▵t_{n}=▵t$ yielding

$$C_{n\to n+1}=-\frac{\partial V}{\partial q_{i,n}}▵t$$

and thus

(15) $$C_{n\to n+1}\propto -\frac{\partial V}{\partial q_{i,n}}=F_{i,n}$$

with a constant time step and causality being of variable nature.

## 3. Consequences of a Discrete Time: On the Origin of the Second Law of Thermodynamics

While there are many consequences of a discrete time such as a maximal acceleration [12] or the cosmic constant and the inflation of the universe [6], the focus here is the second law of thermodynamics. The basic microscopic physical laws are time reversible and only the second law of thermodynamics, which is a macroscopic physical representation of the world, is able to describe irreversible processes in an isolated system through the change of entropy ▵S>0 [12,13]. In 2014, we showed that at the microscopic level time irreversibility can be obtained if time is of discrete nature [5] applying the discrete dynamical time concept of Lee introduced above [11]. It was further demonstrated that the consequences of a continuous time description of physics if time is actually of discrete origin is a microscopic entropy, which upon ensemble averaging is equal to the Clausius entropy and Boltzmann/Gibbs entropy under a slow changing force limit. With other words, entropy is an artifact when describing a macroscopic system under a continuous time. This microscopic entropy of a *Z* particle system at state *n*, which can easily be made macroscopic by proper averaging, is thereby described by the following equation:

(16) $$S_{n}=-\sum_{i=1}^{Z}N_{i,df}\;k_{B\;}\;ln\;s_{i,n}$$

with $k_{B}$ the Boltzmann constant and $N_{i,df}$ the number of degree of freedoms of particle *i* and $s_{i,n}$ the time scaling factor that follows the reversibility axiom $\frac{s_{n+1}}{s_{n}}=\frac{F_{x,n}}{F_{x,n+1}}$ (assuming only a change of the force along the *x*-axis for simplicity). The entropy change from causality event *n* to n+1 is then given by [5]

(17) $$▵S=S_{n+1}-S_{n}=\sum_{i=1}^{Z}N_{i,df}\;k_{B\;}\;ln\;\frac{F_{x,i,n}}{F_{x,i,n+1}}$$

Upon ensemble averaging this description yields the Boltzmann entropy under a slow changing force limit [4].

The presented description of time being the metric of causality described through the force $F_{i,n}=\left(F_{x,i,n},0,0\right)$ is now further elaborated on using Equation (15) yielding

(18) $$▵S=S_{n+1}-S_{n}=\sum_{i=1}^{Z}N_{i,df}\;k_{B\;}\;ln\;\frac{C_{i,n\to n+1}}{C_{i,n+1\to n+2}}$$

Equation (18) establishes the correlation between entropy change and causal event change under the assumption that time is the metric of causality yielding the following second law of thermodynamics

(19) $$▵S=S_{n+1}-S_{n}=\sum_{i=1}^{Z}N_{i,df}\;k_{B\;}\;ln\;\frac{C_{i,n\to n+1}}{C_{i,n+1\to n+2}}\geq 0$$

in an isolated system with equality if the system is in thermodynamic equilibrium. This results in the following entropy definition at time point *n*.

(20) $$S_{n}=-\sum_{i=1}^{Z}N_{i,df}\;k_{B\;}\;ln\;C_{i,n\to n+1}$$

It is noted that this is an entropy of a system under study contrasting the Boltzmann entropy, which is an ensemble averaged entity. In order to compare the two we shall describe the two entropies of a large system of Z independent, identical, indistinguishable particles (such as the ideal gas or diluted gas) for which each micro-state has the same probability. Boltzmann showed that the entropy is then given by

(21) $$S^{B}=-k_{B}\;<ln\;p_{j}>=-k_{B}Z\sum_{j=1}^{J}p_{j\;}ln\;p_{j}$$

with $p_{j}=\frac{Z_{j}}{Z}$ being the probability of a single particle to be in the state *j* and the average is a single particle average taken over all the possible states *J* of the particle.

In comparison, following reference [5] the ensemble averaged microscopic entropy of this Z particles system under a slow changing force limit is given by $<S_{n}>\approx -k_{B\;}Z\sum_{l=1}^{J}\;p_{l}\;ln\;\left(p_{l}\;s_{l,n}\right)$. Following this formula, the ensemble averaged causality-derived entropy of Eqution (20) is then given by

(22) $$<S_{n}>\approx -k_{B\;}Z\sum_{l=1}^{J}p_{l\;\;}ln\left(p_{l\;}\;C_{l,n\to n+1}\right)\approx -k_{B\;}Z\sum_{l=1}^{J}p_{l\;\;}ln\;p_{l\;}=S^{B}$$

Thus, for a system with independent non-interacting particles in the “slow causality limit” with $C_{l,n\to n+1}$, the average microscopic entropy approximates the Boltzmann entropy of the system. An interpretation of this analogy suggests that the original description of the entropy by Boltzmann is just a statistical averaging of a variable which is close to a constant (i.e., $C_{l,n\to n+1}$) but that the entropy term itself has not its root in statistical mechanics but in causality.

### 3.1. The Relationship between Causality and Entropy Discussed by Gedankenexperimente

In this paragraph Equations (19) and (20) are discussed on four well known thermodynamic examples and the universe to illustrate the action of causality through the microscopy entropy derived.

**Figure 1:**
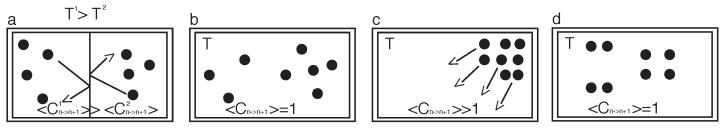
Gedankenexperimente (**a**) An isolated system (indicated by double walls) is composed of two closed sub-systems with unequal temperature that can exchange heat through the single wall. The systems are filled with an ideal gas. The gas molecules indicated by spheres from the left sub-system (1) have a larger kinetic energy yielding a larger momentum change in average at the heat exchanging wall than the molecules from the right sub-system (2) when hitting the wall yielding a larger causality on the left sub-system when compared with the right sub-system (**b**) An ideal gas system in equilibrium. (**c**) A system in which the ideal gas molecules are concentrated on the right top corner. (**d**) The system is in thermodynamic equilibrium and a special state is selected.

(i) Let us first elaborate on the classical example with two temperature reservoirs with $T_{1}>T_{2}$ that can exchange heat and only heat while the entire system is isolated (Figure 1a). This is an important Gedankenexperiment because it has been shown by Planck that this example of two heat exchanging reservoirs can be translated into any other thermodynamic process that undergoes an irreversible process towards the thermodynamic equilibrium [13]. Following the second law of thermodynamics this system goes into the thermodynamic equilibrium with a temperature $T_{eq}$ with $T_{1}>T_{eq}>T_{2}$. Following the causality argument, it is evident that the temperature bath with $T_{1}$ comprises gas particles with a higher kinetic energy yielding on average a larger force (by repulsion because of a large momentum change at the wall) and correspondingly a larger average causality at the heat exchanging wall than the particles from the $T_{2}$ reservoir. Part of these forces via causality dissipate through the wall with a net transfer from $T_{1}$ to $T_{2}$ (exchanging thereby heat) until an equilibrium is obtained.

(ii) The next Gedankenexperiment is an isolated system in equilibrium (Figure 1b). If an isolated system is in the thermodynamic equilibrium from a macroscopic perspective no causal events happen since the system is in equilibrium and hence ▵S=0 with F=0 with *F* the force of the entire system as it is observed macroscopically. It is evident that from a microscopic perspective there are many causal events (for example in a container filled with ideal gas molecules in the thermodynamic equilibrium there are constant collisions and along with it acting forces, while macroscopically no measurable changes occur). However, the causal events do not dissipate into smaller ones. At the thermodynamic limit (with Z→∞) at each time step overall the same causal relationships occur inside the isolated system and with it the same local acting forces $F_{x,i,n}$ when summed over all molecules and thus the microscopic entropy of Equation (20) do not change in average and with it also not the macroscopic one [5].

(iii) This description brings us to an example of having an ideal gas at a given temperature in an isolated container localized in one corner of the container with a directed velocity (Figure 1c). According to Boltzmann, this is a rather unique state of the system and the entropy increases until the gas molecules are distributed evenly in the container, which is the most probably state of the system. In accordance, the causal events of the gas molecules dissipate into smaller ones because at the beginning a concerted force of the gas molecules is present such that the gas molecules may fly into the wall in a concerted fashion yielding a large force, which dissipates into smaller ones after many collisions until the causality is approaching 1.

(iv) Next, the ideal gas in equilibrium is studied and a unique case is selected in which the observer defines a single equilibrium micro-state out of the many as special (Figure 1d). In the approach of Boltzmann, this system is unique and an entropy increase is expected, which makes no sense because the system is in equilibrium. This apparent paradox can be solved by requesting ensemble averaging to be mandatory because the unique selected micro-state will never reoccur. As a consequence, Boltzmann entropy is only applicable on a system for which many repetitive experiments have been done. In the concept presented here the sum of the acting forces to all the molecules in the system is always independent on the micro-state selected and thus the causal entropy will not change as expected since the system is in equilibrium. The microscopic entropy in Equation (20) is thus independent of the observer and does not request a macroscopic view and thus gets rid at least of some of the paradox that come along with the Boltzmann entropy [14].

(v) We end with a Gedankenexperiment on the evolution of the universe using Newtonian physics for simplification [6]. The causal event with the largest acting force ever was at the beginning of the universe in the big bang also requesting the smallest time step. Causal events are dissipating since into smaller ones (in part because the universe expands) along with longer and longer time steps yielding an arrow of time described by Equation (19). It is interesting to note that this description of the second law of thermodynamics is valid for a single universe at any time event, while the classical Clausius one or the statistical one by Boltzmann require a temperature bath outside the isolated universe, a universe that evolves from equilibrium to equilibrium in infinitely slow steps, and ensemble averaging in the latter theory. Furthermore, the non-trivial request of having at the big bang the universe in the most ordered state with the lowest entropy according to Boltzmann [15] gets obvious with Equation (20) because at the beginning the causal events had the largest acting forces.

### 3.2. In Quantum Mechanics There Is No Time

The relationship of Equation (20) between causality and entropy requests the presence of causality. However, causality is absent in standard quantum mechanics [1]. If the derived relationship between causality and entropy is thus only true for non quantum mechanical systems on the first notion it appears to be a limited concept requesting a discussion. For this, we first need to clarify the terms causality and time in the context of standard quantum mechanics [1,16]. The non-causal nature of quantum mechanics can be exemplified by a measurement of a quantum mechanical system which outcome is not determined for a single experiment most popularly visualized by the Schrödinger cat [16], which is alive and dead at the same time before one has a look at it and upon measurement can be either alive or dead. Actually, quantum mechanics lacks causality by definition since a quantum mechanical system can have superimposed states (such as alive and dead in the example of the cat). From a causality point of view superimposed states means that a system is always covering all the possible states simultaneously and thus no causal chain is eminent. Next, time must be discussed within quantum mechanics. It must be thereby noted that there is no time in quantum mechanics reflected by the lack of a time operator. The time-dependent Schrödinger equation comprises a classical time and is thus of semi-classical nature [16] with a time therein that comes from an adjacent classical clock [17,18], the latter which of course follows causality as discussed above. Thus, the time evolution of the wave function described by the time-dependent Schrödinger equation pinpoints to the time evolution of the classical outcome being of causal nature. The non-causal property of quantum mechanics culminates in its projection to the classical world via the Born rule in the measurement. However, the statistical ensemble of measurements described by the Born rule is of classical nature and thus can cause an effect along with time as the metric yielding also to an entropy. The Born rule can therein be used to establish a non-unitary process building a basis for entropy [19]. The von Neumann entropy is such an entropy, which is of classical nature as it is not an operator. It builds upon the density operator, which is a statistical entity ensemble averaged over all the states and thus not time sensitive. With other words, time as well as entropy are treated classically in quantum mechanics and thus the established relationship between entropy and causality described in Equation (20) holds also for standard quantum mechanics. From the rather different perspective of the transactional interpretation, which describes a measurement as a collapse between a retarded and advanced wave function an arrow of time in quantum mechanics can also be obtained by using a causal argument that requests also a discrete time (in addition to a discrete space) [19,20].

## 4. Discussion

The nature of time is one of the big mysteries both in physics as well as in philosophy. While Aristoteles states time measures change [3], Newton states that it ticks [7]. Aristoteles mentioned further that we measure time through a change of motion and motion through time indicating thereby how its definition slips one through the fingers. While in general relativity theory time is the fourth dimension along with the three space coordinates, it is evident that a human being, while able to move forward and backward in space, cannot do so in time. Augustinus therefore distinguishes between the present, the past in the present and the future in the present, highlighting the fact that time is different from space coordinates as we can only be in the present. Our body can measure the consequence of a force which means causality. If time is the metric of causality it is thus reasonable that we can only be in the present and neither in the past nor the future. Our body is thereby a typical device, which can only measure if there is an effect from a cause. This is also true for a clock, which is the device of time measurement. A clock is of periodic nature requesting a momentum change and with it a force and actually a changing force causing clock to be of causal nature. By introducing time as the metric of causality the usual delineation between time and causality is reversed as has been proposed by Reichenbach [4] with the consequence that time is no longer a fundamental physical entity. Actually, a system is defined by the coordinate and momenta (q,p) and their gradients along space and causality arises from there. This can be exemplified by an object that travels with a constant velocity. Since nothing happens, there is no causality and thus no time. Somebody may point out that there is velocity and thus time must be taken into account because velocity is the first time derivative of space. However, since the reference frame of the system can be put to the object, the system can also be described in absence of a velocity and thus no time is needed to describe the system as long as there is not an acting force and correspondingly causality. Of course, this is an idealization and in nature there is always a cause and an effect because gravitation acts everywhere; it is omni-present. So, each causal event is related to other ones and as time is the metric of causality it is ubiquitous (which does not mean that it is fundamental). The time step sizes between the events are however dependent on the causal system and the choice of the reference frame and are therefore relative in nature yielding thereby for example time dilation as demonstrated in special relativity, but this accounts only for the time step size and not the acclaimed fundamental causal numbering. If it is in the interest of the experimenter to compare two distinct systems in respect to causal events, they are best compared each to a third system that comprises a higher frequency of events, which is called a clock.

Following this description on the causal chain it is evident that it comprises an arrow of time. This can be exemplified with the evolution of the universe starting with the big bang being the strongest causal event followed by the “dissipation” into smaller and more events (because the universe expands) towards a state where nothing will happen anymore (if the universe will not collapse eventually), which is nothing but the thermodynamic equilibrium of the universe. In the established physics theories an arrow of time is obtained only with the second law of thermodynamics through the entropy (while indirectly). By defining here time as the metric of causality and demonstrating its discrete nature a relationship between entropy change and causality change is established suggesting the origin of entropy to be causality with an entropy increase when the causal chain dissipates into smaller causal events. This interpretation appears to contrast the Boltzmann approach in its root because it is not about the measure of disorder but the measure of order. However, of course these two entities are related with each other directly. In this context, it is mentioned that recently another approach to a derivation of entropy has been proposed that is based on the cognition between past and future in the brain relating the two entities also with a chain of information [21].

Finally, it is interesting to elaborate on the discrete nature of time. It has been demonstrated here that time must be quantized if it is the metric of causality. Another approach for defining time recently introduced by Lucia and Grisolia by using the ratio between entropy and entropy rate also yielded a discrete nature of time [22]. Discrete time physics is fascinating and reveals many interesting insights into fundamentals of physics [23,24,25,26,27,28,29,30]. Furthermore, with time to be discrete and space continuous, time and space albeit both variables of the space-time vector get inherently distinct as requested from the notion of an arrow of time prohibiting traveling backward in time and the free motion in space including both directions forward and backward.

## 5. Conclusions

In summary, the present work introduced time as the metric of causality. With this approach, causality through the second law of Newton within classical physics is fundamental, and time is only the metric. With the time as the metric of causality, time is requested to be of discrete nature yielding an entropy description from the consequence of causal changes. The reader is invited to elaborate further on the many consequences of a discrete time in physics.
